# Rational Design of Porous N-Ti_3_C_2_ MXene@CNT Microspheres for High Cycling Stability in Li–S Battery

**DOI:** 10.1007/s40820-019-0341-6

**Published:** 2019-12-12

**Authors:** Jianli Wang, Zhao Zhang, Xufeng Yan, Shunlong Zhang, Zihao Wu, Zhihong Zhuang, Wei-Qiang Han

**Affiliations:** School of Materials Science and Engineering, Zhejiang University, 38 Zheda Road, Hangzhou, 310027 People’s Republic of China

**Keywords:** Spray drying method, N-Ti_3_C_2_ MXene@CNT microspheres, Nitrogen-doping, High cycling stability, Lithium–sulfur battery

## Abstract

Herein, N-Ti_3_C_2_@CNT microspheres are successfully synthesized by the simple spray drying method. In the preparation process, HCl-treated melamine (HTM) is selected as the sources of carbon and nitrogen. It not only realizes in situ growth of CNTs on the surface of MXene nanosheets with the catalysis of Ni, but also introduces efficient N-doping in both MXene and CNTs. Within the microsphere, MXene nanosheets interconnect with CNTs to form porous and conductive network. In addition, N-doped MXene and CNTs can provide strong chemical immobilization for polysulfides and effectively entrap them within the porous microspheres. Above-mentioned merits enable N-Ti_3_C_2_@CNT microspheres to be ideal sulfur host. When used in lithium–sulfur (Li–S) battery, the N-Ti_3_C_2_@CNT microspheres/S cathode delivers initial specific capacity of 927 mAh g^−1^ at 1 C and retains high capacity of 775 mAh g^−1^ after 1000 cycles with extremely low fading rate (FR) of 0.016% per cycle. Furthermore, the cathode still shows high cycling stability at high C-rate of 4 C (capacity of 647 mAh g^−1^ after 650 cycles, FR 0.027%) and high sulfur loading of 3 and 6 mg cm^−2^ for Li–S batteries.

**Electronic supplementary material:**

The online version of this article (10.1007/s40820-019-0341-6) contains supplementary material, which is available to authorized users.

## Highlights

- N-Ti_3_C_2_@CNT microspheres are successfully synthesized by the simple spray drying and one-step pyrolysis.
- Within the microsphere, MXene nanosheets intimately interact with CNTs constructing porous and highly conductive network, which can provide strong immobilization for polysulfides.
- N-Ti_3_C_2_@CNT microsphere/S cathode shows highly cycling stability in lithium-sulfur battery.

**Electronic supplementary material:**

The online version of this article (10.1007/s40820-019-0341-6) contains supplementary material, which is available to authorized users.

## Graphical abstract

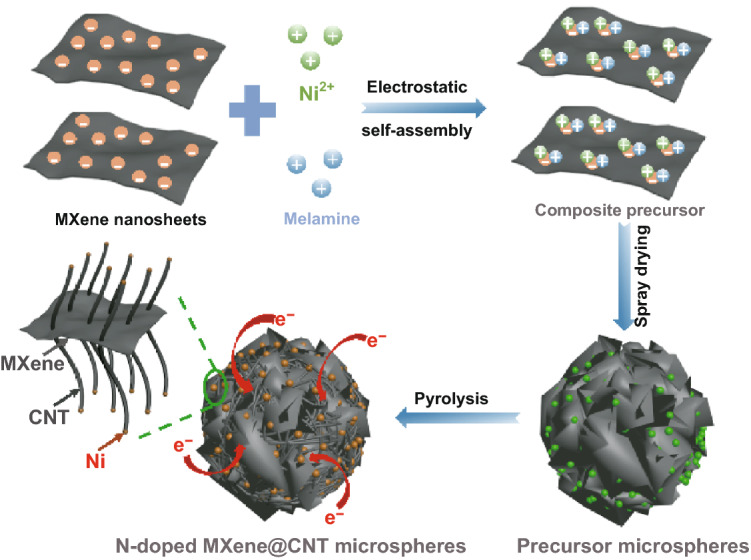

## Introduction

Lithium–sulfur (Li–S) battery is considered as one of the most promising next-generation second batteries due to higher energy density (2600 Wh kg^−1^) and theoretical capacity (1672 mAh g^−1^) compared with conventional lithium ion batteries. And sulfur is environmentally benign and naturally abundant [1, 2]. However, the practical development of Li–S battery still faces some obstacles. Firstly, sulfur is inherently insulating, which causes inferior capacity performance and poor rate capability. In addition, huge volume change (up to 80%) results in the destruction of cathode structure during cycles. Furthermore, the shuttle effect originated from the dissolution of lithium polysulfides (LiPSs) in electrolytes leads to rapid capacity fading and poor cycle life [3, 4]. To overcome above problems, extensive efforts have been performed including the employment of sulfur host materials [5], the functionalization of separators [6], and the introduction of solid-state electrolytes [7]. Among these strategies, employing host materials is simple and effective one to promote cathode performance. And lots of researches focused on exploring new materials as ideal sulfur host, which should preferably possess porous structure, excellent electronic conductivity, and strong immobilization for LiPSs [8, 9].

Carbon-based materials (porous carbon, carbon nanotubes, graphene oxide, etc.) were widely applied as sulfur hosts because of good conductivity and abundant porosity [10]. Unfortunately, the physical adsorption for LiPSs is too weak to effectively suppress shuttle effect, indicating unsatisfied cycling stability. More studies paid attention to polar metal oxides and metal sulfides, which provide stronger chemical anchoring for LiPSs [11, 12]. However, these materials (like MnO_2_, TiO_2_) usually suffer from intrinsically poor conductivity, which causes sluggish electrochemical kinetics and finally leads to unsatisfied capacity performance and rate capability.

Nazar et al. [13] firstly employed exfoliated MXene as sulfur host. It was found that MXene can provide strongly chemical immobilization for LiPSs via Lewis acid–base affinity. MXene is a large series consisted of ternary metal carbides/nitrides and denoted by M_*n*+1_X_*n*_T_*x*_, where M is a transition metal such as Ti or Mo, X is carbon and/or nitrogen, and T are surface functional groups such as –OH and –F. Since Gogotsi et al. [14] firstly reported MXene in 2011, various MXenes have been identified. MXenes have been widely investigated as the electrode materials due to excellent conductivity, high specific surface area, and good flexibility [15, 16].

MXene is considered as a promising sulfur host in Li–S battery owing to above-mentioned advantages. However, MXene nanosheets tend to restack on account of hydrogen bonds, which lowers active area and hinders full utilization in Li–S battery [17]. Incorporating another material with MXene to fabricate composite is an effective strategy to solve above problems [18]. In recent years, the composites of MXene/TiO_2_ [19], MXene/reduce graphene oxide (rGO) [20, 21], MXene/MoS_2_ [22], and MXene/carbon nanotubes (CNTs) [23, 24] have been reported and showed promoted electrochemical performance compared with single MXene. Nazar et al. [25] prepared interwoven MXene/CNT composites as an effective sulfur host. The additive of CNTs not only prevented aggregation of MXene, but also further improved conductivity of composite. The Lewis acidic affinity and thiosulfate/polythionate conversion jointly formed the dual immobilization for LiPSs, resulting in high cycling stability [26]. However, the direct mixing of MXene and CNTs faces the dispersion problem, and the interaction is inferior. Cheng et al. [27] successfully prepared MXene@CNT composites through CVD strategy, in which CNTs uniformly dispersed on the surface of MXene sheets. However, CVD method is involved in harsh conditions. Zhang et al. [28] synthesized Co-CNT/MXene composites originating from ZIF-67/MXene for the oxidation–reduction reaction (ORR). Fast electron transfer could be attributed to strong contact between MXene and CNTs via in situ growth of CNTs. But the preparation of ZIF is complicated and hard to control. In addition, the MXene/CNT composites with sphere-like structure are still not reported.

Herein, we synthesized N-Ti_3_C_2_@CNT composites via a facile method. In the preparation process, HCl-treated melamine (HTM) was decomposed to realize in situ growth of CNTs on MXene nanosheets at high temperature and introduce nitrogen-doping. Furtherly, N-Ti_3_C_2_@CNT microspheres were successfully prepared through the spray drying, followed by one-step pyrolysis. Within the microsphere, N-Ti_3_C_2_ MXene interacted with N-CNTs to form a 3D well-interconnected porous network, which facilitated fast electron/ion transfer in electrodes. Nitrogen-doping could not only improve electronic conductivity, but also promote anchoring capability for LiPSs. As the sulfur host in Li–S battery, N-Ti_3_C_2_@CNT microspheres/S cathode demonstrated high specific capacity of 1339.2 mAh g^−1^ at 0.1 C, excellent rate capability, and superior long cycling stability (capacity decay rate of only 0.016% per cycle after 1000 cycles at 1 C). Good performance was derived from the virtues of microspheres: (1) high specific surface area and abundant porosity provided space to accommodate active sulfur and offered more adsorption sites to anchor LiPSs; (2) efficient incorporation of MXene and CNTs facilitated electron transport across the microspheres, which enhanced electrochemical reaction kinetics; (3) highly polar MXene and N-doping effectively anchored LiPSs within the microspheres, resulting in highly stable cycling performance. The strategy can also be extended to prepare other CNT microsphere composites and shows great potential in the field of energy storage.

## Experimental Section

### Preparation of Ti_3_C_2_ MXene Nanosheets

Firstly, Ti_3_AlC_2_ (Forsman Technology (Beijing) Co., Ltd) powders were dispersed in hydrofluoric acid (HF, 40 wt%) solution and vigorously stirred for 48 h at room temperature. The suspension was centrifuged and washed in deionized (DI) water for several times. After vacuum dried at 60 °C, multilayered Ti_3_C_2_ MXene was obtained. To prepare MXene nanosheets, 1.2 g above-obtained Ti_3_C_2_ MXene was poured into 20 mL tetrabutylammonium hydroxide (TMAOH, 25 wt%) and stirred at room temperature for 24 h. After centrifugation and washing with DI water, the precipitates were dispersed in 200 mL DI water and sonicated for 2 h under Ar atmosphere. The suspension was centrifuged for 1 h at 3500 rpm. The dark green supernatant was collected and stored at 4 °C, which consisted of Ti_3_C_2_ MXene nanosheets.

### Preparation of N-Ti_3_C_2_, N-Ti_3_C_2_@CNTs, and N-Ti_3_C_2_@CNT Microspheres

NiCl_2_ and HCl-treated melamine were mixed in a certain amount of Ti_3_C_2_ nanosheets solution to obtain suspension A. The precipitates were collected by centrifugation and then freeze-dried for 12 h. The powders were placed in the alumina boat and pyrolyzed at 800 °C for 2 h with the heating rate of 2 °C min^−1^ under Ar condition. The as-received product was designated as N-Ti_3_C_2_@CNTs. As comparison, N-Ti_3_C_2_ was prepared by the similar process but from absence of NiCl_2_. Furtherly, the composite precursors were prepared through the spray drying from suspension A. The powders were heated to 800 °C for 2 h under Ar condition to finally receive N-Ti_3_C_2_@CNT microspheres.

### Preparation of Sulfur Composite Materials

The sulfur composite materials were obtained via the melting diffusion strategy. Generally, the host material and sulfur were mixed with the weight ratio of 3:7 and placed in Teflon-lined stainless steel autoclave. After 155 °C for 12 h, the sulfur composite materials can be received.

### Materials Characterization

Scanning electron microscopy (SEM, Hitachi SU-8010) and transmission electron microscopy (TEM, JEM-2100) measurements were taken to estimate the morphology and internal structure of samples. X-ray diffraction (XRD) patterns were obtained by Rigaku MiniFlex 600 (Cu Kα radiation). Thermogravimetric analysis (TGA) was performed by the simultaneous thermal analysis instrument (METTLER TOLEDO TGA/DSC 3+). Micromeritics ASAP 2020 Plus HD88 was employed to measure N_2_ adsorption–desorption curves. The surface chemistry was analyzed based on X-ray photoelectron spectroscopy (XPS) in Thermo Fisher Scientific Escalab 250Xi.

### Electrochemical Measurements

Electrochemical performances were evaluated based on 2032-type coin cells. The sulfur composite, super P, and LA132 were dispersed in deionized water with stirring. The as-obtained slurry was uniformly spread on the surface of carbon-coated Al foil. After vacuum-dried at 60 °C overnight, the cathode plate was obtained. The sulfur loading of cathode is ca. 1.5 mg cm^−2^ if no special introduction. The assembly process of cells was conducted in Ar-filled glove box with the sulfur cathode, lithium foil, separator, and liquid electrolyte. The electrolyte is composed of 1 M lithium bis(trifluoromethane)sulfonimide (LiTFSI) in a mixture of 1,3-dioxolane/1,2-dimethoxyethane (DOL/DME, v/v, 1:1) with the additive of 2 wt% LiNO_3_. Solartron 1400 electrochemical workstation was employed to obtain CV curves and electrochemical impedance spectroscopy (EIS) spectra. The measurement of CV curves was taken at the scanning rate of 0.1 mV s^−1^ with the voltage window of 1.8–2.8 V. Galvanostatic charge/discharge was conducted by the LAND-CT2001 instrument at various C-rates (1 C = 1672 mA g^−1^) with potential range from 1.8 to 2.8 V.

## Results and Discussion

**Fig. 1:**
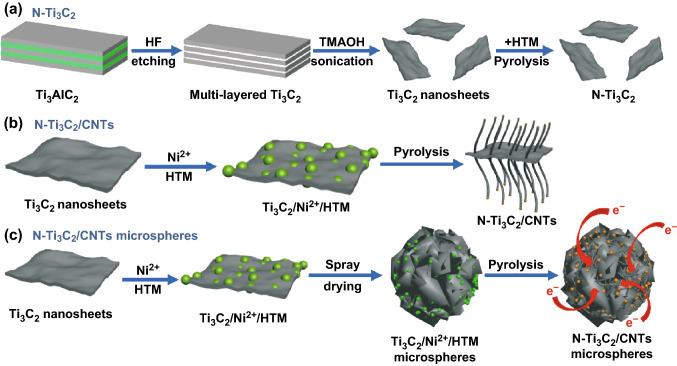
Schematic illustration of preparation of **a** N-Ti_3_C_2_, **b** N-Ti_3_C_2_@CNTs, and **c** N-Ti_3_C_2_@CNT microspheres

Figure 1 illustrates the synthesis process of N-Ti_3_C_2_ MXene, N-Ti_3_C_2_@CNTs, and N-Ti_3_C_2_@CNT microspheres. Firstly, Ti_3_C_2_ MXene nanosheets were obtained after etching of hydrofluoric acid and further sonication exfoliation. Ni^2+^ and HCl-treated melamine (HTM) were selected as the catalyst precursor and carbon source, respectively, which can be effectively adsorbed on the surface of negatively charged MXene sheets via the electrostatic force [29, 30]. As displayed in Fig. S1, uniform dark green solution indicates successful delamination of multilayered Ti_3_C_2_. When positively charged Ni^2+^ and/or HTM suspension were mixed with above solution, the obvious precipitates and a clear supernatant can be observed. The process is involved with electrostatic interaction between MXene sheets and Ni^2+^/HTM. Finally, porous N-Ti_3_C_2_@CNT microspheres are synthesized through the spray drying and pyrolysis. During the pyrolysis, Ni^2+^ was reduced to Ni to achieve the in situ growth of CNTs on the surface of MXene. Meanwhile, N-rich HTM was decomposed to introduce N-doping in the CNTs and Ti_3_C_2_. As comparison, N-Ti_3_C_2_ MXene and N-Ti_3_C_2_@CNTs were prepared by the route of Fig. 1.

Figure S10 shows XRD patterns of multilayered Ti_3_C_2_ and Ti_3_C_2_ nanosheets. After sonication exfoliation, the (0002) diffraction peak of Ti_3_C_2_ shifts to 6.3° from 9.1° and the intensity increases, demonstrating effective exfoliation of multilayered Ti_3_C_2_ [31]. Compared with Ti_3_C_2_ sheets, the peak of N-Ti_3_C_2_ MXene shifts by 0.2° to 6.1°, which indicates the existence of N-doping in MXene structure [30, 32]. N-dopants increase interlayer spacing of MXene layers due to larger atomic radius of nitrogen than carbon element, leading to slight shift of peak. The peaks located at 36.2°, 42.4°, and 61.5° belong to Ti_3_C_2_ MXene and no peaks of anatase or rutile type TiO_2_ can be observed, demonstrating good stability of MXene in the synthesis process [33]. For N-Ti_3_C_2_@CNTs and N-Ti_3_C_2_@CNT microspheres, the additional peaks can be attributed to (002) plane of graphitic carbon (26.3°) and nickel (44.4° and 51.8°), respectively [34]. The results demonstrate successful synthesis of CNTs with the catalysis of Ni species. The XRD patterns (Fig. S14) of sulfur composites verify successful infiltration of orthorhombic sulfur by the simple melting diffusion strategy. TGA was performed to evaluate sulfur proportion of sulfur composites. As shown in Fig. S15, the obvious mass loss from 180 to 350 °C was resulted from evaporation of sulfur under N_2_ atmosphere [35]. The sulfur content can be calculated as 71.6, 71.7, and 71.8% in N-Ti_3_C_2_/S, N-Ti_3_C_2_@CNTs/S, and N-Ti_3_C_2_@CNT microspheres/S, respectively.

**Fig. 2:**
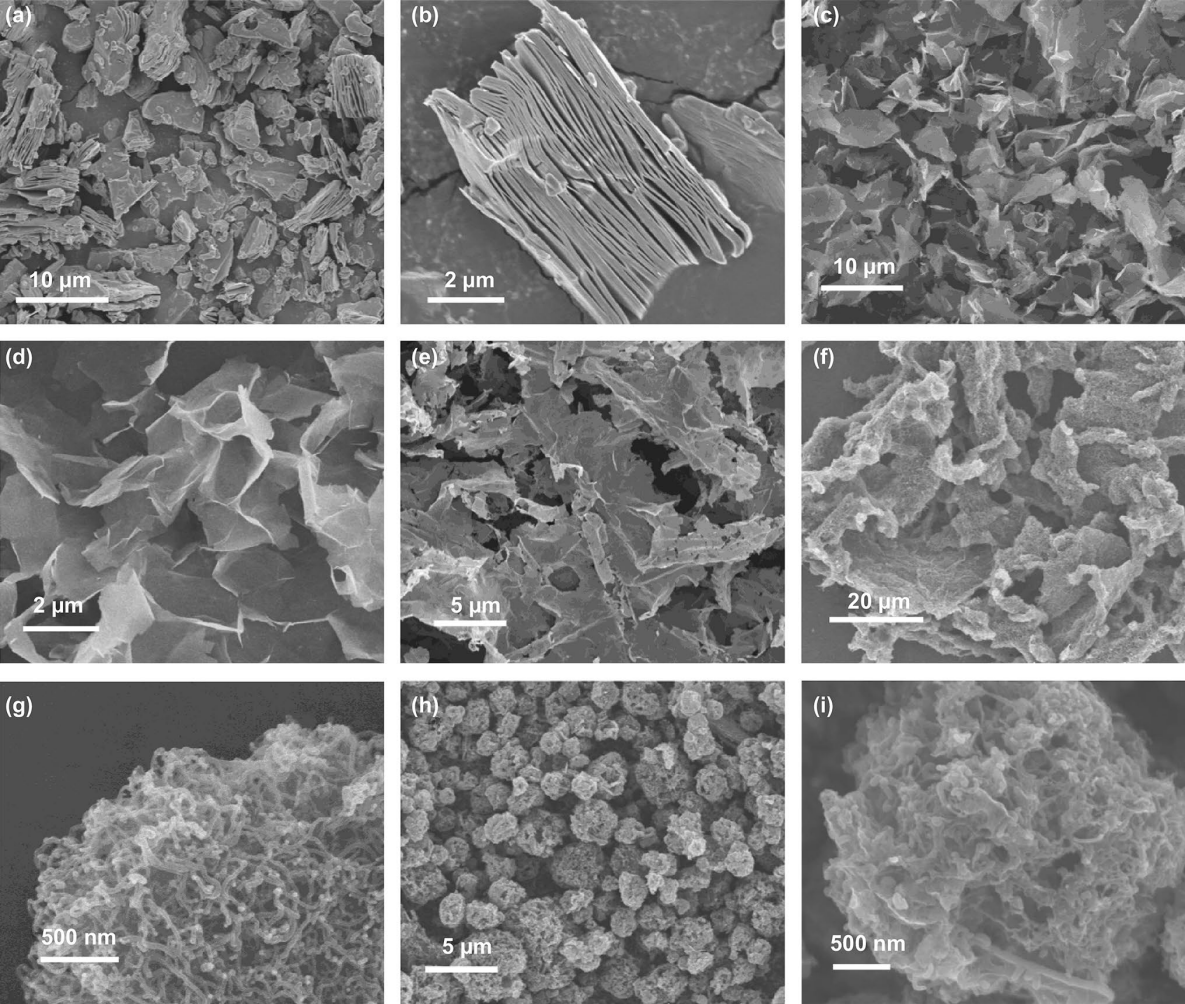
SEM images of **a**, **b** multilayered Ti_3_C_2_ MXene after etching, **c**, **d** Ti_3_C_2_ MXene nanosheets after exfoliation, **e** N-Ti_3_C_2_, **f**, **g** N-Ti_3_C_2_@CNTs, and **h**, **i** N-Ti_3_C_2_@CNT microspheres

**Fig. 3:**
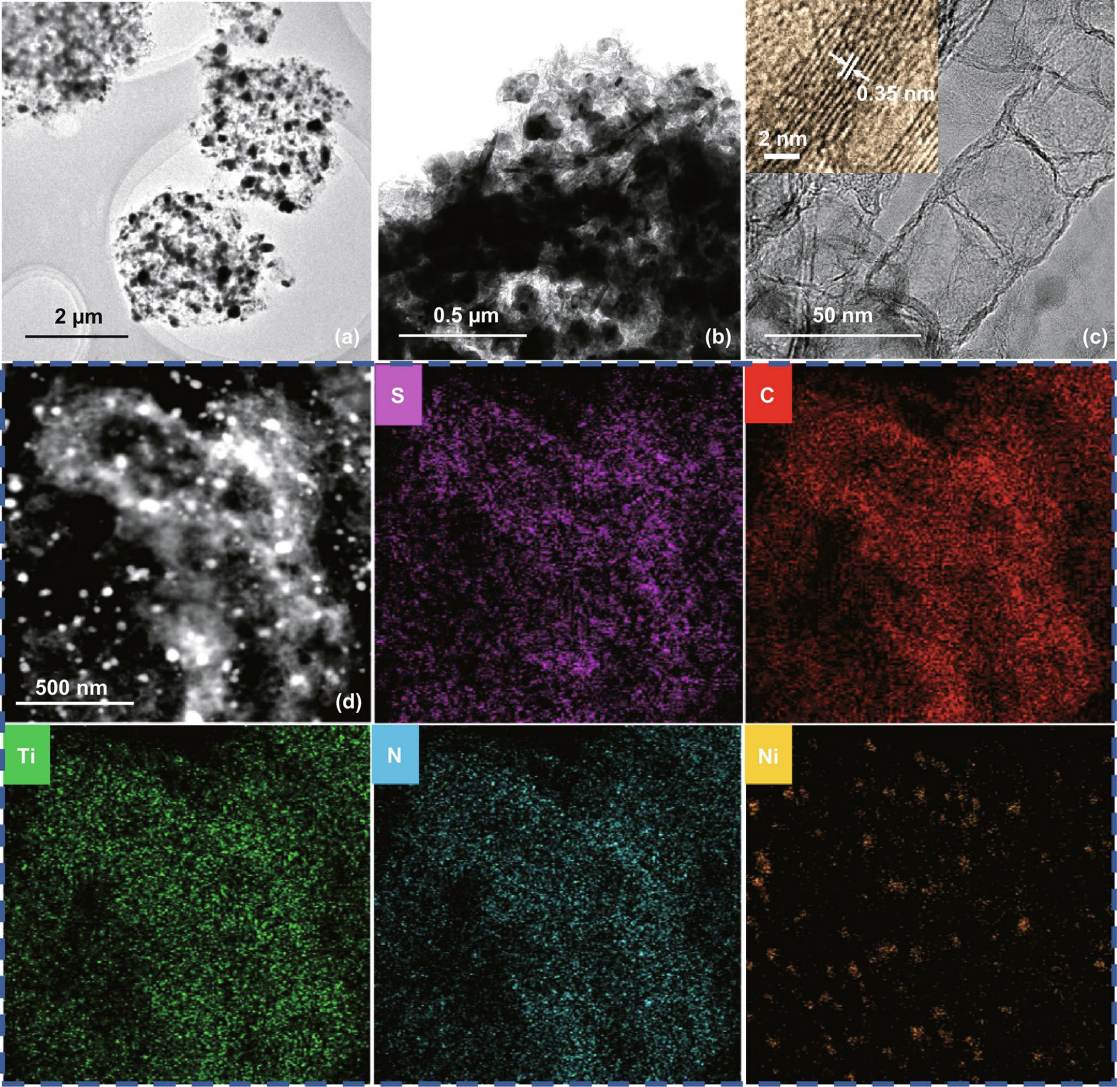
**a**–**c** TEM and HRTEM images of N-Ti_3_C_2_@CNT microspheres and **d** EDS elemental mapping of N-Ti_3_C_2_@CNT microspheres/S composite

SEM and TEM were conducted to identify the morphology and structure of as-obtained materials. Multilayered Ti_3_C_2_ MXene (Fig. 2a, b) shows typical accordion-like structure, demonstrating successful removal of Al layers. After sonication, MXene possesses nanosheet-like morphology with lateral sizes of several micrometers (Fig. 2c, d). Crumpled surface reveals good flexibility and ultrathin thickness, indicating successful preparation of high-quality MXene nanosheets. TEM images (Fig. S2) show transparent flake-like structure for Ti_3_C_2_ MXene nanosheets, indicating good exfoliation effect. N-Ti_3_C_2_ (Fig. 2e) still maintains micro-sized sheet-like structure with distinct macropores on the surface. TEM images (Fig. S3) obviously reveal nano-sized micropores and mesopores in the N-Ti_3_C_2_, which can be attributed to high temperature and reduction atmosphere produced by decomposition of melamine [36, 37]. The hierarchical pores are favorable to promote specific surface area and pore volume. As shown in Fig. S5, the precursors are composed of porous microspheres after spray drying. After pyrolysis, N-Ti_3_C_2_@CNT microspheres still retain uniform sphere-like morphology with particle size of 2–5 μm (Fig. 2h, i). TEM images (Fig. 3a–c) obviously reveal that N-Ti_3_C_2_@CNT microsphere consists of interconnected CNTs and MXene nanosheets. In addition, some Ni nanoparticles are homogenously distributed within the microsphere. The small amount of Ni particles can not only improve electronic conductivity, but also provide chemical confinement for polysulfides [38]. CNTs show bamboo-like structure with fringes spacing of 0.35 nm corresponding to (002) planes [39], illustrating high graphitization degree of CNTs with the catalysis of Ni. EDS mapping (Fig. S6) testifies homogenous distribution of CNTs and MXene in the porous microsphere. For N-Ti_3_C_2_@CNTs, CNTs are successfully synthesized and uniformly distributed on the surface of MXene to form interwoven porous morphology (Figs. 2f, g and S4).

After sulfur encapsulation, N-Ti_3_C_2_@CNTs and N-Ti_3_C_2_@CNT microspheres (Fig. S7c–f) still maintain previous structure and no distinct sulfur aggregation can be seen, indicating successful infiltration of sulfur into porosity of microspheres. However, N-Ti_3_C_2_ and sulfur aggregate together to form clusters (Fig. S7a, b). And EDS mapping (Fig. S8) reveals that sulfur aggregation exists in the clusters. The result demonstrates the structure advantage of N-Ti_3_C_2_@CNTs and N-Ti_3_C_2_@CNT microspheres in accommodating sulfur compared with N-Ti_3_C_2_ nanosheets. As displayed in Fig. 3d, sulfur has similar intensity within N-Ti_3_C_2_@CNT microsphere, indicating uniform distribution of sulfur. Furthermore, elements of Ti, C, N, and Ni also homogenously dispersed in the region, which furtherly confirm that CNTs and MXene are uniformly distributed in the microsphere.

N_2_ adsorption/desorption measurement was taken to evaluate porous structure of materials. Ti_3_C_2_ nanosheets have higher specific surface area (*SSA*) and pore volume (*PV*) than that of multilayered Ti_3_C_2_ (Fig. S11), demonstrating effective exfoliation. After pyrolysis with HTM, SSA, and PV of MXene (N-Ti_3_C_2_) significantly increased to 263.3 m^2^ g^−1^ and 0.43 cm^3^ g^−1^ (Fig. S12), respectively. The isotherm curves of N-Ti_3_C_2_@CNTs and N-Ti_3_C_2_@CNT microspheres (Figs. S13 and 4b) exhibit typical IV-type behavior with obvious hysteresis loop, which can be attributed to the existence of mesopores and macropores [40]. In addition, the presence of micropores is evidenced by the rapid increase in N_2_ absorption when the relative pressure is close to zero [41]. N-Ti_3_C_2_@CNTs and N-Ti_3_C_2_@CNT microspheres possess high *SSA* of 358.4, 388.6 m^2^ g^−1^ and PV of 0.66, 0.72 cm^3^ g^−1^, respectively, which are much higher than that of N-Ti_3_C_2_. The increased part can be assigned to the incorporation of MXene flakes and CNTs. The abundant porosity not only allows infiltration of enough sulfur, but also effectively buffers volume expansion of electrodes. In addition, porous structure is favorable to electron/ion transformation and permeation of liquid electrolytes [42].

**Fig. 4:**
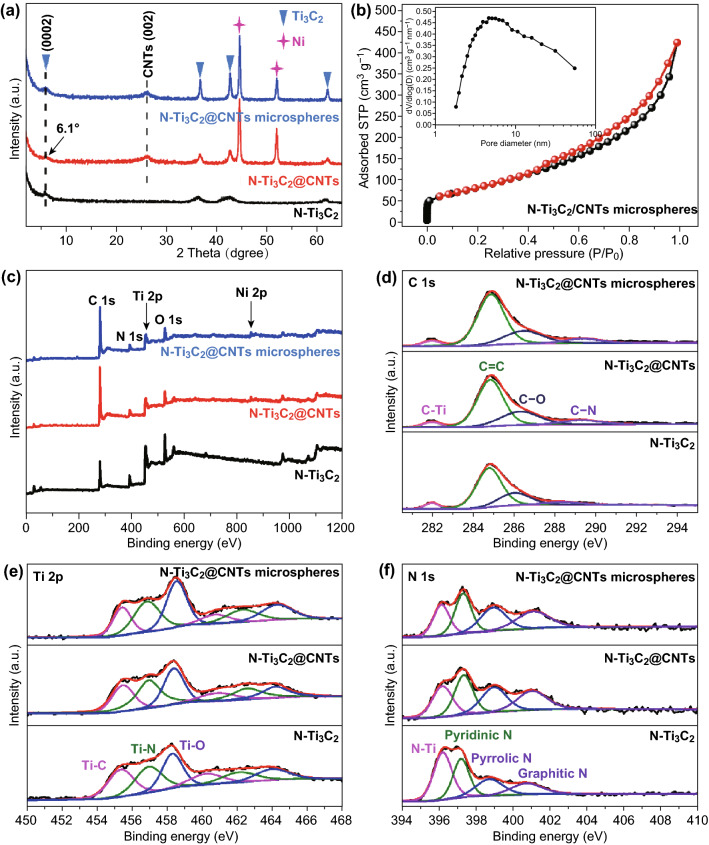
**a** XRD patterns of three kinds of materials, **b** N_2_ adsorption/desorption isotherm curves and pore size distribution of N-Ti_3_C_2_@CNT microspheres, **c** XPS survey, **d** C 1s spectra, **e** Ti 2p spectra, and **f** N 1s spectra of N-Ti_3_C_2_, N-Ti_3_C_2_@CNTs, and N-Ti_3_C_2_@CNT microspheres

X-ray photoelectron spectroscopy (XPS) was performed to analyze elements content and condition. Figure 4c reveals the existence of C, Ti, N, and O elements in three kinds of materials. Especially, the peak of Ni can be observed in N-Ti_3_C_2_@CNTs and N-Ti_3_C_2_@CNT microspheres, which catalyze in situ growth of CNTs. The Ti 2p spectra can be identified to three peaks located at 455.5, 456.9, and 458.5 eV (Fig. 4e), which corresponds to Ti–C, Ti–N, and Ti–O, respectively. The appearance of Ti–N bonding demonstrates N-doping in Ti_3_C_2_ structure. The N-Ti bonding in N 1s spectra (Fig. 4f) also verifies the existence of nitrogen dopants [32]. In addition to N–Ti peak, another three peaks centered at 397.4, 399.1, and 401.0 eV can be assigned to pyridinic N, pyrrolic N, and graphitic N [43, 44]. Pyridinic N is dominated among three kinds of N element, which is considered to be main immobilization sites of LiPSs for N-doped materials [45]. Compared with N-Ti_3_C_2_, the decrease in N-Ti in N-Ti_3_C_2_@CNTs can be ascribed to the presence of N-doping in CNTs structure. High-resolution C 1s spectra (Fig. 4d) consist of C–Ti, C=C, C–O, and C–N bodings. It can be noted that the peak intensity of C–N of N-Ti_3_C_2_@CNTs and N-Ti_3_C_2_@CNT microspheres is significantly higher than that of N-Ti_3_C_2_, which reconfirms the existence of N-doped CNTs. N-doping exists in both of MXene and CNTs according to above results. The nitrogen content of N-Ti_3_C_2_ MXene, N-Ti_3_C_2_@CNTs, and N-Ti_3_C_2_@CNT microspheres is up to 16.48, 10.98, and 11.86 at% (Table S2). The efficient N-doping can not only promote electronic conductivity, but also improve the immobilization ability for LiPSs [29, 34].

**Fig. 5:**
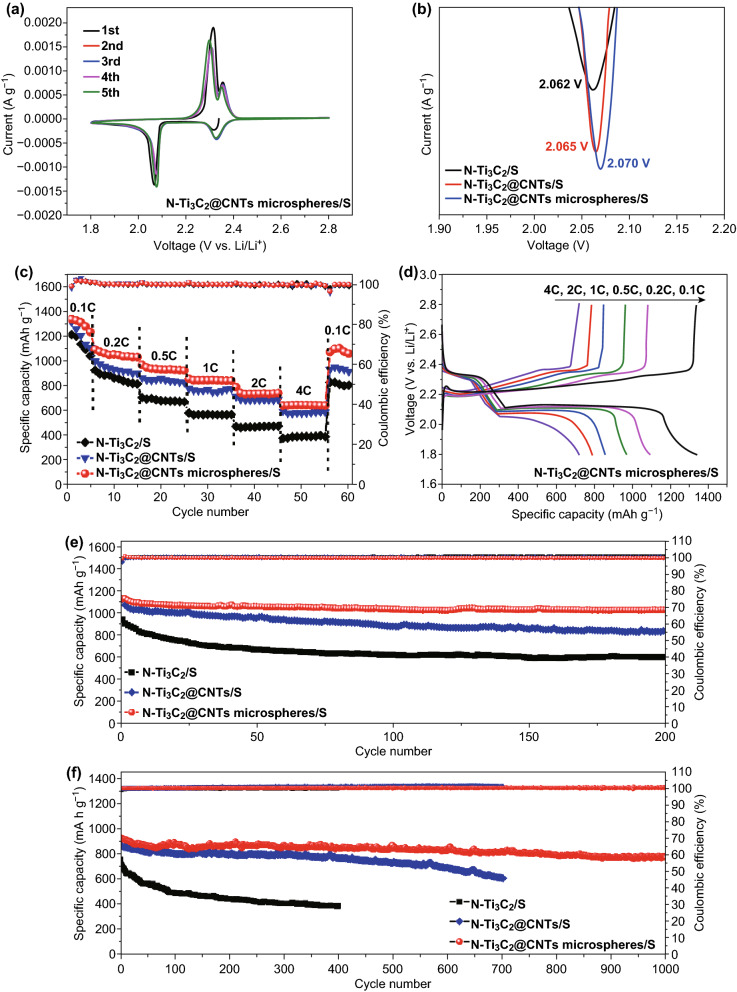
**a** CV curve of N-Ti_3_C_2_@CNT microspheres/S cathode at the scanning rate of 0.1 mV s^−1^. **b** Magnification of cathodic peaks (voltage range of 1.9–2.2 V). **c** Rate capability of three cathodes at different C-rate (the calculation of specific capacity is based on the weight of sulfur in the cathode). **d** The galvanostatic charge/discharge curves for N-Ti_3_C_2_@CNT microspheres/S cathode at various C-rates. Cycling performance of three cathodes at **e** 0.2 C and **f** 1 C

The CV curves of three sulfur cathodes (Figs. 5a and S16) exist two well-defined cathodic peaks and one sharp anodic peak. The former peaks correspond to the reduction process from sulfur to long-chain lithium polysulfides (LiPSs) and furtherly to Li_2_S_2_ and Li_2_S. The latter peak is associated with reversed transformation of above process [46–48]. It can be noted that the second reduction peak of N-Ti_3_C_2_@CNT microspheres/S cathode is higher than that of N-Ti_3_C_2_@CNTs/S and significantly higher than N-Ti_3_C_2_/S cathode (Fig. 5b). The result demonstrates that N-Ti_3_C_2_@CNT microspheres exhibit more favorable structure in facilitating electron/ion transformation [49]. The galvanostatic charge/discharge curves of N-Ti_3_C_2_@CNTs/S (Fig. S17b) and N-Ti_3_C_2_@CNT microspheres/S (Fig. 5d) cathodes show obvious discharge/charge plateaus even at high C-rate of 4 C (1 C = 1672 mA g^−1^). However, the plateaus for N-Ti_3_C_2_/S nearly disappear at high current density (Fig. S17a). The difference can be attributed to the incorporation of CNTs into MXene, which effectively improves electronic conductivity leading to faster electrochemical kinetics. Figure 5c shows the rate capability. N-Ti_3_C_2_@CNT microspheres/S cathode delivers reversible specific capacity of 1339.2, 1093.3, 969.4, 856.3, 789.3, and 640.5 mAh g^−1^ at C-rate of 0.1, 0.2, 0.5, 1, 2, and 4 C, respectively. High specific capacity of 1103.2 mAh g^−1^ can be retained when C-rate decreases to 0.1 C, demonstrating excellent rate capability [50]. The capacity performance is better than that of N-Ti_3_C_2_@CNTs/S cathode and much better than N-Ti_3_C_2_/S cathode.

**Fig. 6:**
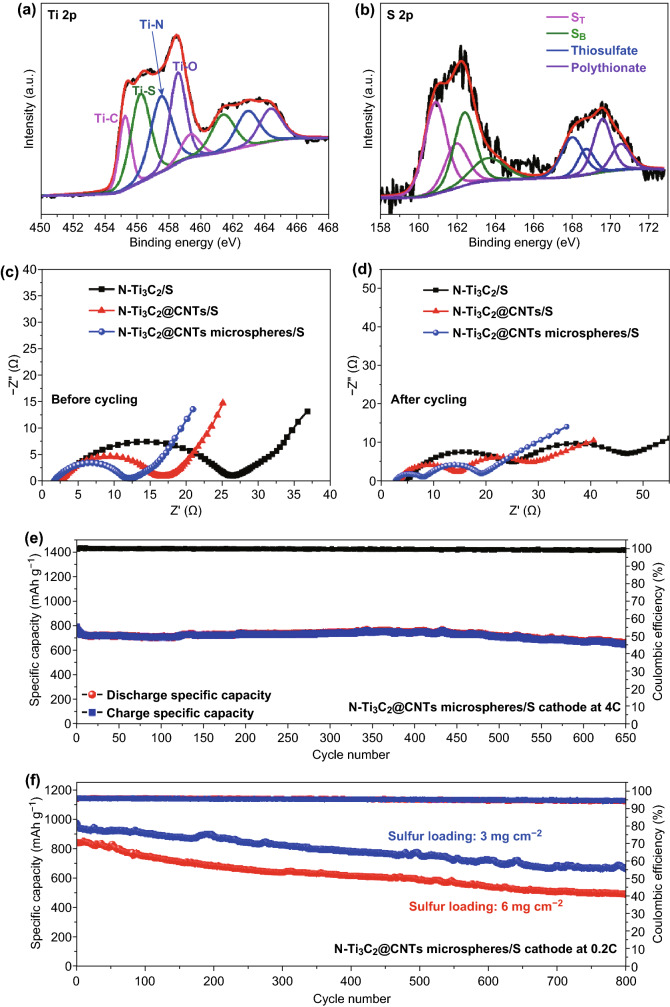
**a** Ti 2p and **b** S 2p spectra of N-Ti_3_C_2_@CNT microspheres/Li_2_S_6_. EIS spectra of sulfur cathodes **c** before and **d** after cycles. Cycling performance of N-Ti_3_C_2_@CNT microspheres/S cathode at **e** high C-rate of 4 C and **f** high sulfur loading

The cycling performances at 0.2 C were investigated and are displayed in Fig. 5e. N-Ti_3_C_2_@CNT microspheres/S cathode exists initial capacity of 1124.6 mAh g^−1^ and reversible capacity of 1025.3 mAh g^−1^ after 200 cycles corresponding to high capacity retention (*CR*) up to 91.2%, which is significantly higher than that of N-Ti_3_C_2_/S cathode (598.5 mAh g^−1^ after 200 cycles, CR 63.6%). N-Ti_3_C_2_@CNTs/S cathode also shows good cycling stability with capacity of 835.9 mAh g^−1^ after 200 cycles and CR of 74.7%. The difference of cycling stability becomes more distinct at 1 C (Fig. 5f). The specific capacity of N-Ti_3_C_2_/S cathode decreases to only 358.5 mAh g^−1^ after 400 cycles with low cycling stability. N-Ti_3_C_2_@CNTs/S cathode exhibits specific capacity of 874.9 mAh g^−1^ and maintains capacity of 604.5 mAh g^−1^ after 700 cycles with low capacity fading rate (FR) of 0.044% per cycle. N-Ti_3_C_2_@CNT microspheres/S cathode shows highly stable cycling performance up to 1000 cycles. Particularly, the cathode delivers initial capacity of 927.5 mAh g^−1^ and reversible capacity of 775.6 mAh g^−1^ after 1000 cycles with extremely low FR of only 0.016% per cycle. Good cycling stability is remarkable compared with reported studies (Table S3). EIS was investigated to clarify improved electrochemical performance. The plots show one depressed semicircle and a sloped line before cycling (Fig. 6c), which correspond to charge transfer resistance (*R*_ct_) and charge diffusion resistance (*W*), respectively [51]. *R*_ct_ orderly diminishes from N-Ti_3_C_2_/S and N-Ti_3_C_2_@CNTs/S to N-Ti_3_C_2_@CNT microspheres/S cathode, which reveals enhanced conductivity of relevant host materials. Additional semicircle emerges at high-frequency region after cycling (Fig. 6d), which is associated with the resistance of insulating Li_2_S layers (*R*_g_) derived from unconverted part during charge [52]. *R*_g_ and *R*_ct_ of N-Ti_3_C_2_@CNT microspheres/S are lower than that of N-Ti_3_C_2_/S and N-Ti_3_C_2_@CNTs/S, demonstrating an efficient redox process of electrode.

Cycling performance at high C-rate and high sulfur loading was tested and is shown in Fig. 6e, f, respectively. N-Ti_3_C_2_@CNT microspheres/S cathode exhibits high capacity of 788.6 mAh g^−1^ and retains reversible capacity of 647.6 mAh g^−1^ after 650 cycles at high C-rate of 4 C with low fading rate of 0.027% per cycle, indicating excellent cycle stability. At high sulfur loading of 3 mg cm^−2^, a specific capacity of 665 mAh g^−1^ after 800 cycles with low fading rate of 0.039% per cycle can be achieved. As sulfur loading increasing to 6 mg cm^−2^, N-Ti_3_C_2_@CNT microspheres/S cathode still shows highly stable cycling with low decay rate of 0.051% per cycle. Excellent cycling stability can be ascribed to strong immobilization for LiPSs originating from polar surface of MXene and effective N-doping in Ti_3_C_2_ and CNTs.

The adsorption measurement of LiPSs was taken to compare anchoring ability of several host materials (Fig. S22). After standing for only 15 min, the color of containing N-Ti_3_C_2_, N-Ti_3_C_2_@CNTs, or N-Ti_3_C_2_@CNT microspheres becomes shallow compared with the pristine Li_2_S_6_ solution, implying efficient adsorption for LiPSs [53]. However, there is no obvious change for the solution containing CNTs. The difference indicates that polar Ti_3_C_2_ can provide stronger confinement for LiPSs than CNTs. Finally, N-Ti_3_C_2_@CNT microspheres show the best adsorption effect for LiPSs, which can be attributed to porous structure with higher specific surface area to provide more adsorption sites for LiPSs. We also disassembled cells before and after cycling to evaluate change of electrodes. As shown in Fig. S19, the surface of N-Ti_3_C_2_/S cathode was almost covered by a passivation layer after cycling [54, 55]. In contrast, the host materials still maintain pristine morphology even though some passivation layer can be observed for N-Ti_3_C_2_@CNTs/S and N-Ti_3_C_2_@CNT microspheres/S electrodes (Figs. S20, S21). The result is consistent with above discussion. N-Ti_3_C_2_@CNT microspheres not only provide strong immobilization for LiPSs, but also maintain morphology stability during cycling due to highly interwoven structure of MXene and CNTs. In addition, Fig. 6a, b shows the Ti 2p and S 2p spectra of N-Ti_3_C_2_@CNT microspheres/Li_2_S_6_. Compared with pristine N-Ti_3_C_2_@CNT microspheres, there is the typical peak of Ti–S bonding located at 456.1 eV, which can be ascribed to Lewis acid–base interaction originating from polar surface of MXene and polysulfide species [53, 56]. High-resolution S 2p spectra can be deconvoluted to thiosulfate (168.0 eV) and polythionate (169.5 eV) complexes, together with remained polysulfides (S_T_ 160.9 eV, S_B_ 162.4 eV) [25]. The thiosulfate/polythionate species are derived from exposed Ti and hydroxyl groups on the surface of MXene [57].

On the basis of above discussion, excellent electrochemical performance of N-Ti_3_C_2_@CNT microspheres/S cathode can be attributed to the following merits: (1) high specific surface area and pore volume can ensure uniform distribution of sulfur in the microspheres and effectively buffer volume expansion of electrodes during discharging; (2) MXene nanosheets interact with CNTs within the microsphere to construct conductive and porous structure, which improves electrochemical reaction kinetics of electrodes; (3) Ti_3_C_2_ MXene exists Lewis acidic affinity with LiPSs, which effectively relieves shuttle effect resulting in highly stable cycling performance. Especially, porous structure and high N-doping can further promote confinement ability of LiPSs.

## Conclusion

In conclusion, we successfully synthesized porous N-Ti_3_C_2_@CNT microspheres through a facile strategy with low-cost materials (NiCl_2_ and HCl-treated melamine). Melamine, as the source of carbon and nitrogen, not only realizes in situ growth of N-doped CNTs on the MXene nanosheets, but also provides efficient N-doping in the Ti_3_C_2_ structure. Within the microspheres, MXene flakes connect with CNTs constructing highly conductive and porous network to accommodate active sulfur and effectively confine polysulfides. In addition, Ni nanoparticles homogenously distribute within the microspheres, which can furtherly promote conductivity of electrodes and tightly entrap LiPSs in the region of microspheres. Above-mentioned merits make N-Ti_3_C_2_@CNTs microsphere an ideal sulfur host material. When employed as sulfur host in Li–S battery, N-Ti_3_C_2_@CNT microsphere/S cathode shows high capacity performance, excellent rate capability, and long-term cycling stability. Furthermore, superior cycling performance even at high C-rate of 4 C and sulfur loading (3 and 6 mg cm^−2^) can be achieved. Last but not the least, the strategy can be extended to prepare other CNT microsphere composites and shows great potential in the field of energy storage.

## Electronic supplementary material

Below is the link to the electronic supplementary material.

Supplementary material 1 (PDF 1937 kb)

## References

[1] Liu X, Huang JQ, Zhang Q, Mai L (2017). Nanostructured metal oxides and sulfides for lithium–sulfur batteries. Adv. Mater..

[2] Kaiser MR, Ma Z, Wang X, Han F, Gao T (2017). Reverse microemulsion synthesis of sulfur/graphene composite for lithium/sulfur batteries. ACS Nano.

[3] Seh ZW, Sun YM, Zhang QF, Cui Y (2016). Designing high-energy lithium–sulfur batteries. Chem. Soc. Rev..

[4] Rehman S, Khan K, Zhao Y, Hou Y (2017). Nanostructured cathode materials for lithium–sulfur batteries: progress, challenges and perspectives. J. Mater. Chem. A.

[5] Ji XL, Lee KT, Nazar LF (2009). A highly ordered nanostructured carbon-sulphur cathode for lithium–sulphur batteries. Nat. Mater..

[6] Su Y-S, Manthiram A (2012). Lithium–sulphur batteries with a microporous carbon paper as a bifunctional interlayer. Nat. Commun..

[7] Xu R, Yue J, Liu S, Tu J, Han F, Liu P, Wang C (2019). Cathode-supported all-solid-state lithium–sulfur batteries with high cell-level energy density. ACS Energy Lett..

[8] Fang R, Zhao S, Sun Z, Wang D-W, Cheng H-M, Li F (2017). More reliable lithium–sulfur batteries: status, solutions and prospects. Adv. Mater..

[9] Chen L, Shaw LL (2014). Recent advances in lithium–sulfur batteries. J. Power Sources.

[10] Ji LW, Rao MM, Zheng HM, Zhang L, Li YC (2011). Graphene oxide as a sulfur immobilizer in high performance lithium/sulfur cells. J. Am. Chem. Soc..

[11] Rehman S, Guo S, Hou Y (2016). Rational design of Si/SiO_2_@hierarchical porous carbon spheres as efficient polysulfide reservoirs for high-performance Li–S battery. Adv. Mater..

[12] Li Z, Zhang J, Lou XW (2015). Hollow carbon nanofibers filled with MnO_2_ nanosheets as efficient sulfur hosts for lithium–sulfur batteries. Angew. Chem. Int. Ed..

[13] Liang X, Garsuch A, Nazar LF (2015). Sulfur cathodes based on conductive mxene nanosheets for high-performance lithium–sulfur batteries. Angew. Chem. Int. Ed..

[14] Naguib M, Kurtoglu M, Presser V, Lu J, Niu J (2011). Two-dimensional nanocrystals produced by exfoliation of Ti_3_AlC_2_. Adv. Mater..

[15] Boota M, Anasori B, Voigt C, Zhao M-Q, Barsoum MW, Gogotsi Y (2016). Pseudocapacitive electrodes produced by oxidant-free polymerization of pyrrole between the layers of 2D titanium carbide (MXene). Adv. Mater..

[16] Tang X, Zhou D, Li P, Guo X, Wang C, Kang F, Li B, Wang G (2019). High-performance quasi-solid-state mxene-based Li–I batteries. ACS Cent. Sci..

[17] Nan J, Guo X, Xiao J, Li X, Chen W (2019). Nanoengineering of 2D MXene-based materials for energy storage applications. Small.

[18] Tang X, Guo X, Wu W, Wang G (2018). 2D metal carbides and nitrides (MXenes) as high-performance electrode materials for lithium–based batteries. Adv. Energy Mater..

[19] Gao X-T, Xie Y, Zhu X-D, Sun K-N, Xie X-M, Liu Y-T, Yu J-Y, Ding B (2018). Ultrathin mxene nanosheets decorated with TiO_2_ quantum dots as an efficient sulfur host toward fast and stable Li–S batteries. Small.

[20] Song J, Guo X, Zhang J, Chen Y, Zhang C, Luo L, Wang F, Wang G (2019). Rational design of free-standing 3D porous mxene/rGO hybrid aerogels as polysulfide reservoirs for high-energy lithium–sulfur batteries. J. Mater. Chem. A.

[21] Bao W, Xie X, Xu J, Guo X, Song J, Wu W, Su D, Wang G (2017). Confined sulfur in 3D mxene/reduced graphene oxide hybrid nanosheets for lithium–sulfur battery. Chem. Eur. J..

[22] Zhang Y, Mu Z, Yang C, Xu Z, Zhang S (2018). Rational design of mxene/1T-2H MoS_2_–C nanohybrids for high-performance lithium–sulfur batteries. Adv. Funct. Mater..

[23] Zhao M-Q, Ren CE, Ling Z, Lukatskaya MR, Zhang C (2015). Flexible mxene/carbon nanotube composite paper with high volumetric capacitance. Adv. Mater..

[24] Zheng W, Zhang P, Chen J, Tian WB, Zhang YM, Sun ZM (2018). In situ synthesis of CNTs@ Ti_3_C_2_ hybrid structures by microwave irradiation for high-performance anodes in lithium ion batteries. J. Mater. Chem. A.

[25] Liang X, Rangom Y, Kwok CY, Pang Q, Nazar LF (2017). Interwoven mxene nanosheet/carbon-nanotube composites as Li–S cathode hosts. Adv. Mater..

[26] Sim ES, Yi GS, Je M, Lee Y, Chung Y-C (2017). Understanding the anchoring behavior of titanium carbide-based mxenes depending on the functional group in Li–S batteries: a density functional theory study. J. Power Sources.

[27] Li X, Yin X, Han M, Song C, Xu H, Hou Z, Zhang L, Cheng L (2017). Ti_3_C_2_ mxenes modified with in situ grown carbon nanotubes for enhanced electromagnetic wave absorption properties. J. Mater. Chem. C.

[28] Chen J, Yuan X, Lyu F, Zhong Q, Hu H, Pan Q, Zhang Q (2019). Integrating mxene nanosheets with cobalt-tipped carbon nanotubes for an efficient oxygen reduction reaction. J. Mater. Chem. A.

[29] Bao W, Liu L, Wang C, Choi S, Wang D, Wang G (2018). Facile synthesis of crumpled nitrogen-doped mxene nanosheets as a new sulfur host for lithium–sulfur batteries. Adv. Energy Mater..

[30] Jiang H, Wang Z, Yang Q, Tan L, Dong L, Dong M (2019). Ultrathin Ti_3_C_2_T_x_ (mxene) nanosheet-wrapped NiSe_2_ octahedral crystal for enhanced supercapacitor performance and synergetic electrocatalytic water splitting. Nano Micro Lett..

[31] Yu P, Cao G, Yi S, Zhang X, Li C, Sun X, Wang K, Ma Y (2018). Binder-free 2D titanium carbide (mxene)/carbon nanotube composites for high-performance lithium–ion capacitors. Nanoscale.

[32] Wen Y, Rufford TE, Chen X, Li N, Lyu M, Dai L, Wang L (2017). Nitrogen-doped Ti_3_C_2_T_x_ mxene electrodes for high-performance supercapacitors. Nano Energy.

[33] Ma Y, Yue Y, Zhang H, Cheng F, Zhao W (2018). 3D synergistical mxene/reduced graphene oxide aerogel for a piezoresistive sensor. ACS Nano.

[34] Ding Y-L, Kopold P, Hahn K, van Aken PA, Maier J, Yu Y (2016). Facile solid-state growth of 3D well-interconnected nitrogen-rich carbon nanotube-graphene hybrid architectures for lithium–sulfur batteries. Adv. Funct. Mater..

[35] Wang J, Meng Z, Yang W, Yan X, Guo R, Han W-Q (2019). Facile synthesis of rGO/g-C_3_N_4_/CNT microspheres via an ethanol assisted spray-drying method for high-performance lithium–sulfur batteries. ACS Appl. Mater. Interfaces.

[36] Liu J, Zhang H-B, Sun R, Liu Y, Liu Z, Zhou A, Yu Z-Z (2017). Hydrophobic, flexible, and lightweight mxene foams for high-performance electromagnetic-interference shielding. Adv. Mater..

[37] Sang X, Xie Y, Yilmaz DE, Lotfi R, Alhabeb M (2018). In situ atomistic insight into the growth mechanisms of single layer 2D transition metal carbides. Nat. Commun..

[38] Chen T, Cheng B, Zhu G, Chen R, Hu Y (2017). Highly efficient retention of polysulfides in “sea urchin”-like carbon nanotube/nanopolyhedra superstructures as cathode material for ultralong-life lithium–sulfur batteries. Nano Lett..

[39] Cao T, Wang D, Zhang J, Cao C, Li Y (2015). Bamboo-like nitrogen-doped carbon nanotubes with Co nanoparticles encapsulated at the tips: uniform and large-scale synthesis and high-performance electrocatalysts for oxygen reduction. Chem. Eur. J..

[40] Song X, Gao T, Wang S, Bao Y, Chen G, Ding L-X, Wang H (2017). Free-standing sulfur host based on titanium-dioxide-modified porous-carbon nanofibers for lithium–sulfur batteries. J. Power Sources.

[41] Jung DS, Hwang TH, Lee JH, Koo HY, Shakoor RA (2014). Hierarchical porous carbon by ultrasonic spray pyrolysis yields stable cycling in lithium–sulfur battery. Nano Lett..

[42] Park GD, Lee J, Piao Y, Kang YC (2018). Mesoporous graphitic carbon-TiO_2_ composite microspheres produced by a pilot-scale spray-drying process as an efficient sulfur host material for Li–S batteries. Chem. Eng. J..

[43] Yang C, Tang Y, Tian Y, Luo Y, Din MFU, Yin X, Que W (2018). Flexible nitrogen-doped 2D titanium carbides (mxene) films constructed by an ex situ solvothermal method with extraordinary volumetric capacitance. Adv. Energy Mater..

[44] Tian Y, Que W, Luo Y, Yang C, Yin X, Kong LB (2019). Surface nitrogen-modified 2D titanium carbide (mxene) with high energy density for aqueous supercapacitor applications. J. Mater. Chem. A.

[45] Pang Q, Nazar LF (2016). Long-life and high-areal-capacity li s batteries enabled by a light-weight polar host with intrinsic polysulfide adsorption. ACS Nano.

[46] Meng Z, Zhang S, Wang J, Yan X, Ying H (2018). Nickel-based-hydroxide-wrapped activated carbon cloth/sulfur composite with tree-bark-like structure for high-performance freestanding sulfur cathode. ACS Appl. Energy Mater..

[47] Hencz L, Chen H, Ling HY, Wang Y, Lai C, Zhao H, Zhang S (2019). Housing sulfur in polymer composite frameworks for Li–S batteries. Nano-Micro Lett..

[48] Zhang X, Wei Y, Wang B, Wang M, Zhang Y, Wang Q, Wu H (2019). Construction of electrocatalytic and heat-resistant self-supporting electrodes for high-performance lithium–sulfur batteries. Nano-Micro Lett..

[49] Zhou T, Lv W, Li J, Zhou G, Zhao Y (2017). Twinborn TiO_2_–TiN heterostructures enabling smooth trapping-diffusion-conversion of polysulfides towards ultralong life lithium–sulfur batteries. Energy Environ. Sci..

[50] Li X, Wolden CA, Ban C, Yang Y (2015). Facile synthesis of lithium sulfide nanocrystals for use in advanced rechargeable batteries. ACS Appl. Mater. Interfaces.

[51] Liu J, Wei A, Pan G, Xiong Q, Chen F, Shen S, Xia X (2019). Atomic layer deposition-assisted construction of binder-free Ni@N-doped carbon nanospheres films as advanced host for sulfur cathode. Nano-Micro Lett..

[52] Meng Z, Li SJ, Ying HJ, Xu X, Zhu XL, Han WQ (2017). From silica sphere to hollow carbon nitride-based sphere: rational design of sulfur host with both chemisorption and physical confinement. Adv. Mater. Interfaces.

[53] Xiao Z, Li Z, Li P, Meng X, Wang R (2019). Ultrafine Ti_3_C_2_ mxene nanodots-interspersed nanosheet for high-energy-density lithium–sulfur batteries. ACS Nano.

[54] Li Z, Zhang J, Guan B, Wang D, Liu L-M, Lou XW (2016). A sulfur host based on titanium monoxide@carbon hollow spheres for advanced lithium–sulfur batteries. Nat. Commun..

[55] Zheng N, Jiang G, Chen X, Mao J, Jiang N, Li Y (2019). Battery separators functionalized with edge-rich MoS_2_/C hollow microspheres for the uniform deposition of Li_2_S in high-performance lithium–sulfur batteries. Nano-Micro Lett..

[56] Xiao Z, Yang Z, Li Z, Li P, Wang R (2019). Synchronous gains of areal and volumetric capacities in lithium–sulfur batteries promised by flower-like porous Ti_3_C_2_T_x_ matrix. ACS Nano.

[57] Zhang H, Qi Q, Zhang P, Zheng W, Chen J (2019). Self-assembled 3D MnO_2_ nanosheets@delaminated-Ti_3_C_2_ aerogel as sulfur host for lithium–sulfur battery cathodes. ACS Appl. Energy Mater..

