# Supplementary material for: Rational Design of Porous N-Ti3C2 MXene@CNT Microspheres for High Cycling Stability in Li–S Battery
Source: Nanomicro Lett. 2019 Dec 12;12:4. doi: 10.1007/s40820-019-0341-6 (PMC7770867; doi:10.1007/s40820-019-0341-6)
Supplement: Supplementary file 1 — Supplementary material 1 (PDF 1937 kb) [file 40820_2019_341_MOESM1_ESM.pdf]

Supporting Information for

Rational Design of Porous N-Ti_3C_2 MXene@CNT Microspheres for High Cycling Stability Li-S Battery

Jianli Wang[1], Zhao Zhang[1], Xufeng Yan[1], Shunlong Zhang[1], Zihao Wu[1], Zhihong Zhuang[1], Wei-Qiang Han[1,*]

[1]School of Materials Science and Engineering, Zhejiang University, 38 Zheda Road, Hangzhou 310027, People's Republic of China

*Corresponding author. E-mail: hanwq@zju.edu.cn (Wei-Qiang Han)

Supplementary Figures

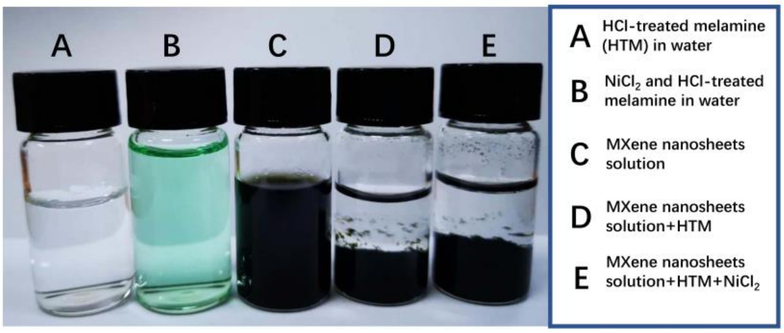

Fig. S1 Digital photographs of MXene nanosheets solution, HTM or (Ni^{2+}+HTM) dissolved in deionized water and self-assembly MXene nanosheets solution via electrostatic force

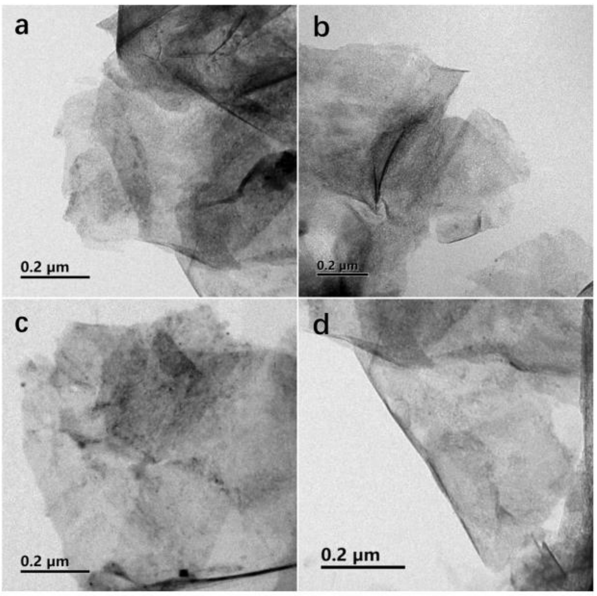

Fig. S2 TEM images of MXene nanosheets after sonication exfoliation

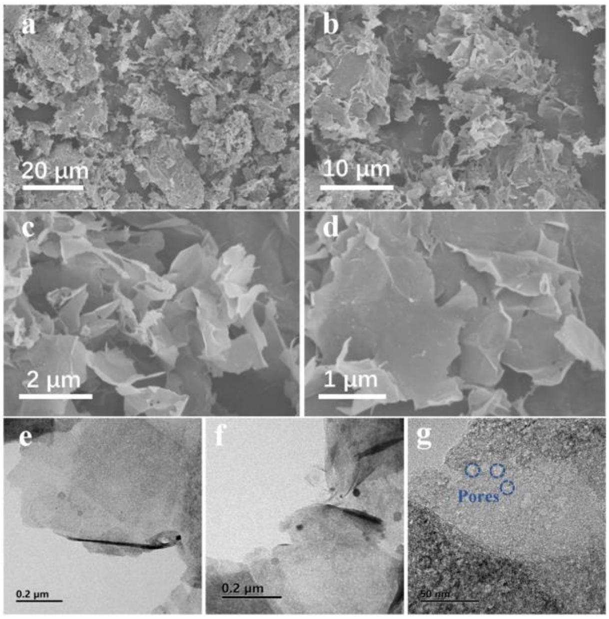

Fig. S3 a-d SEM and **e-g** TEM images of nitrogen-doped Ti_3C_2 MXene nanosheets (N-Ti_3C_2)

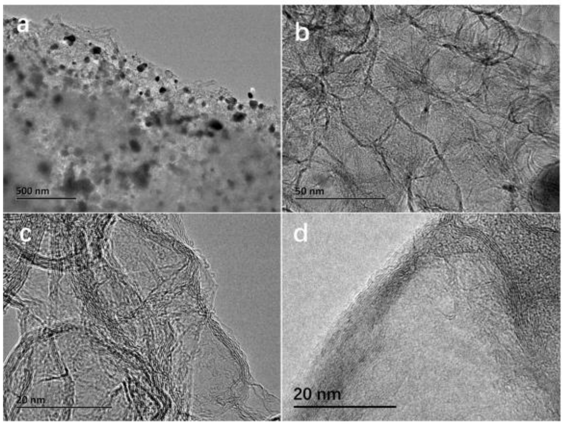

Fig. S4 TEM images of nitrogen-doped Ti_3C_2 nanosheets@CNT composites (N-Ti_3C_2@CNTs)

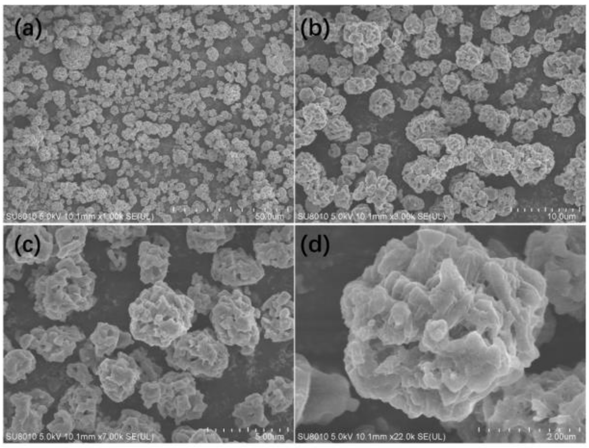

Fig. S5 SEM images of MXene nanosheets/Ni^{2+}/HTM microspheres precursor after the spray drying

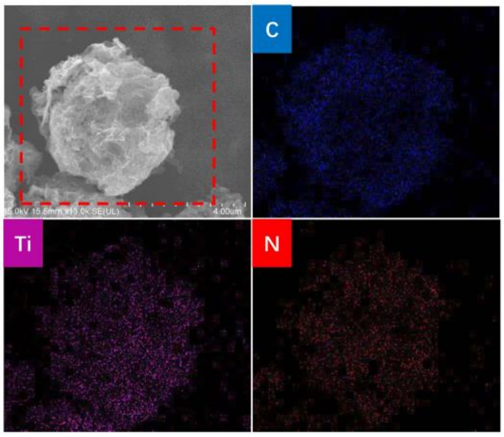

Fig. S6 SEM image and EDS elements mapping of nitrogen-doped Ti_3C_2 nanosheets@CNT microspheres (N-Ti_3C_2@CNT microspheres)

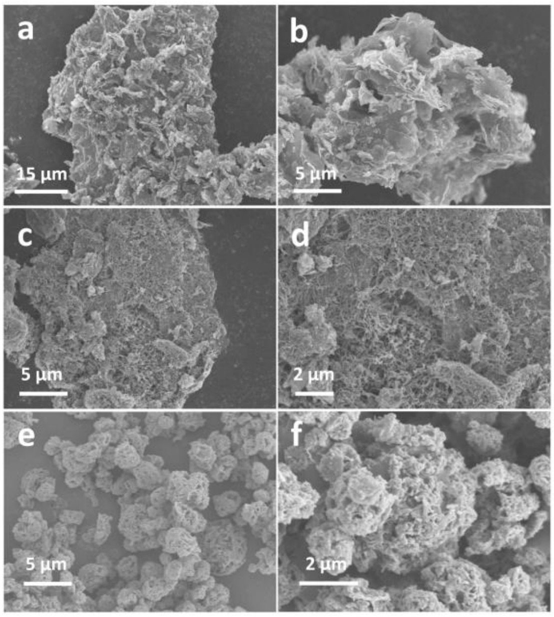

Fig. S7 SEM images of **a, b** N-Ti_3C_2/S, **c, d** N-Ti_3C_2@CNTs/S, and **e, f** N-Ti_3C_2@CNT microspheres/S composites after sulfur infiltration

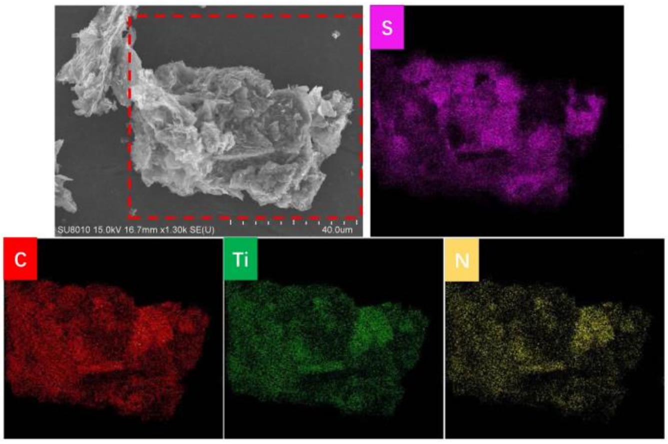

Fig. S8 SEM image and EDS elements mapping of N-Ti_3C_2/S

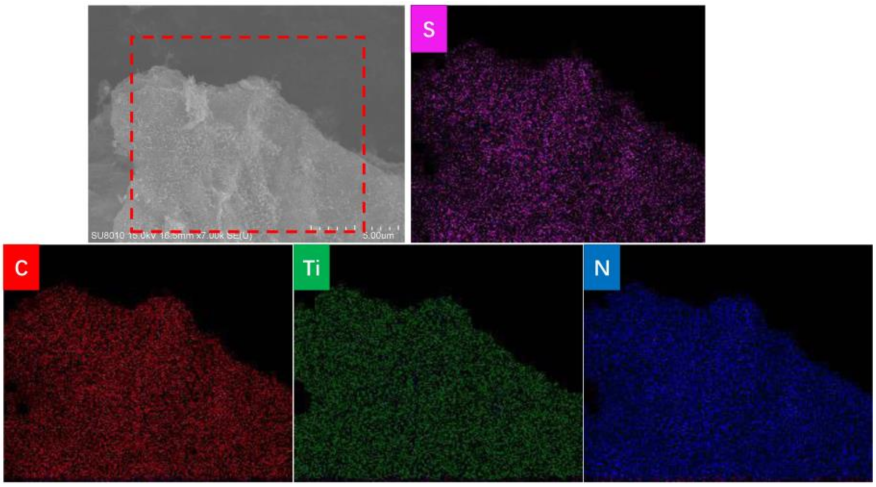

Fig. S9 SEM image and EDS elements mapping of N-Ti_3C_2@CNTs/S

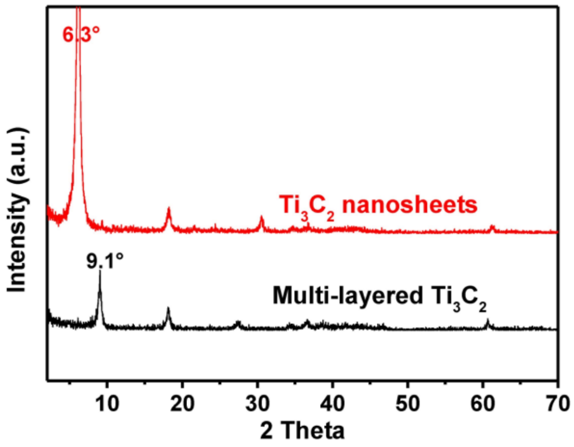

Fig. S10 XRD patterns of multi-layered Ti_3C_2 MXene and Ti_3C_2 nanosheets

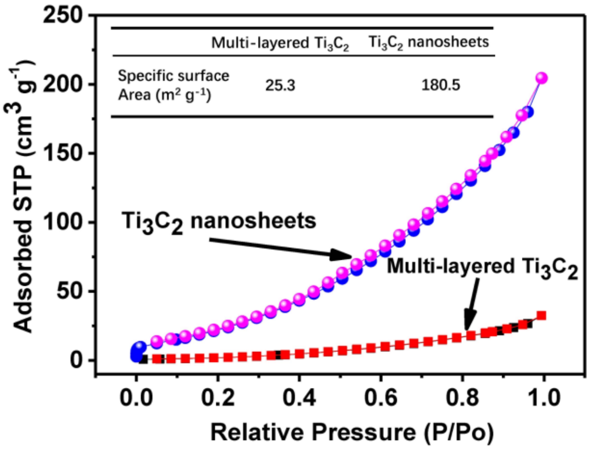

Fig. S11 N_2 adsorption/desorption isotherm curves of multi-layered Ti_3C_2 MXene and Ti_3C_2 nanosheets (inset table: specific surface area)

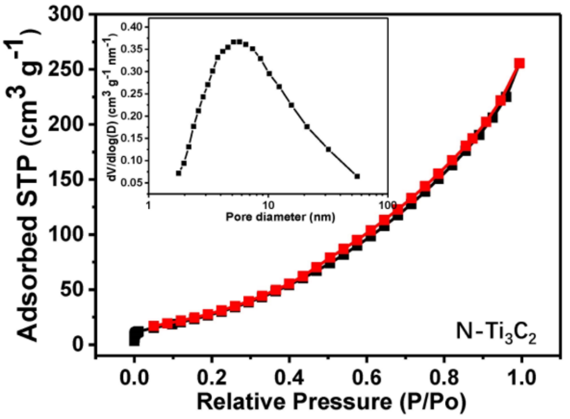

Fig. S12 N_2 adsorption/desorption isotherm curves and pore size distribution of N-Ti_3C_2

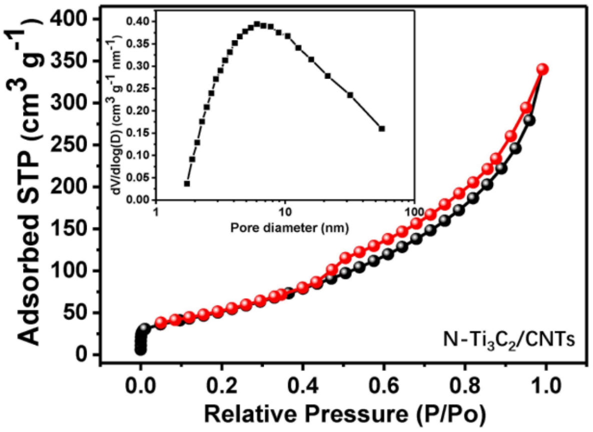

Fig. S13 N_2 adsorption/desorption isotherm curves and pore size distribution of N-Ti_3C_2@CNTs

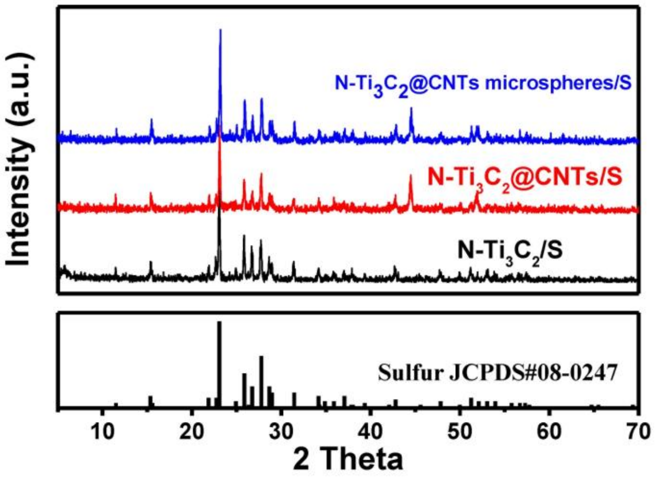

Fig. S14 XRD patterns of N-Ti_3C_2/S, N-Ti_3C_2@CNTs/S and N-Ti_3C_2@CNT microspheres/S composites

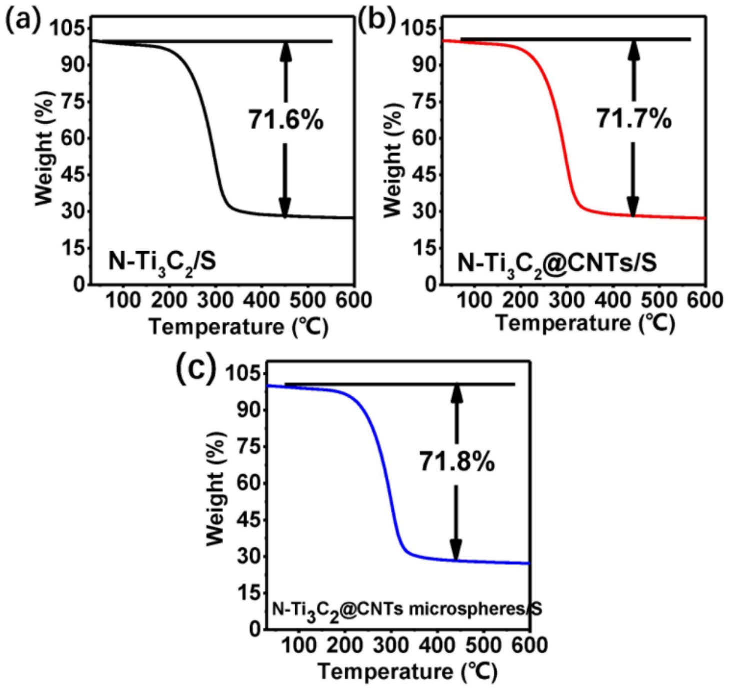

Fig. S15 TGA curves of (**a**) N-Ti_3C_2/S, (**b**) N-Ti_3C_2@CNTs/S and (**c**) N-Ti_3C_2@CNT microspheres/S composites under N_2 atmosphere with heating rate of 10 °C/min

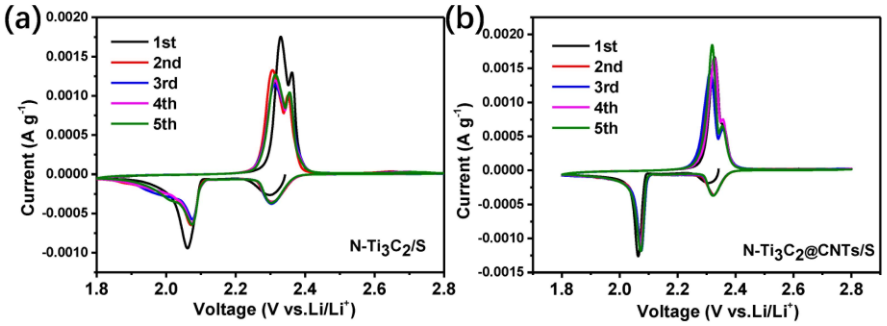

Fig. S16 CV curves of (**a**) N-Ti_3C_2/S and (**b**) N-Ti_3C_2@CNTs/S cathodes at scanning rate of 0.1 mV s^{-1}

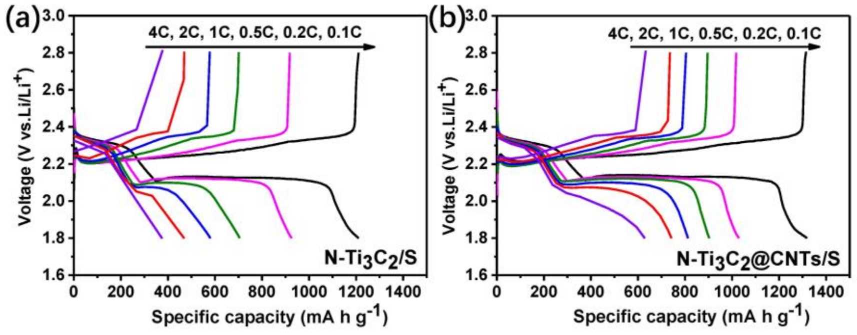

Fig. S17 Charge/discharge profiles of (**a**) N-Ti_3C_2/S and (**b**) N-Ti_3C_2@CNTs/S cathodes at different C-rate

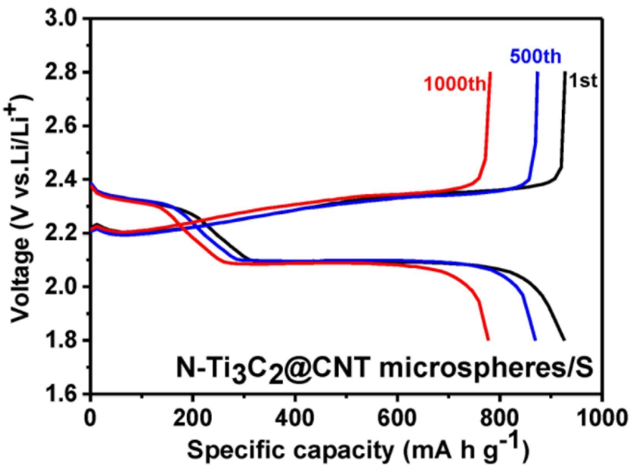

Fig. S18 Charge/discharge profiles of N-Ti_3C_2@CNT microspheres/S cathode for 1st, 500th, and 1000th cycles at 1 C

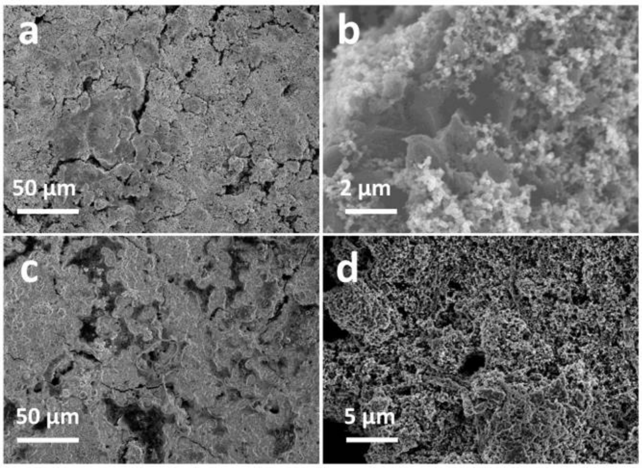

Fig. S19 SEM images of N-Ti_3C_2/S cathode (**a, b**) before and (**c, d**) after 100 cycles at 0.2 C

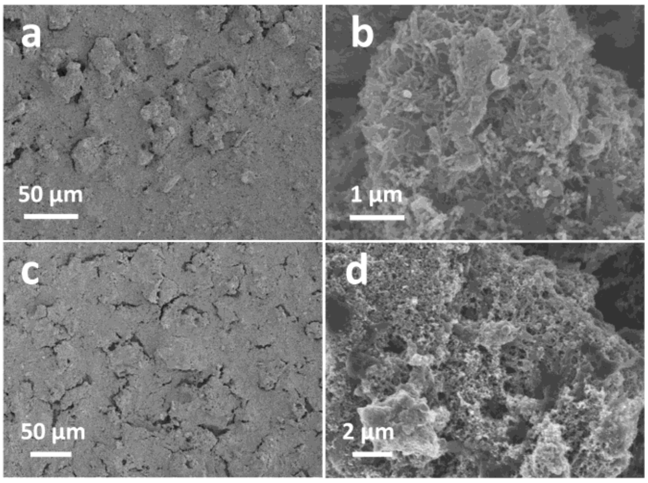

Fig. S20 SEM images of N-Ti_3C_2@CNTs/S cathode (**a, b**) before and (**c, d**) after 100 cycles at 0.2 C

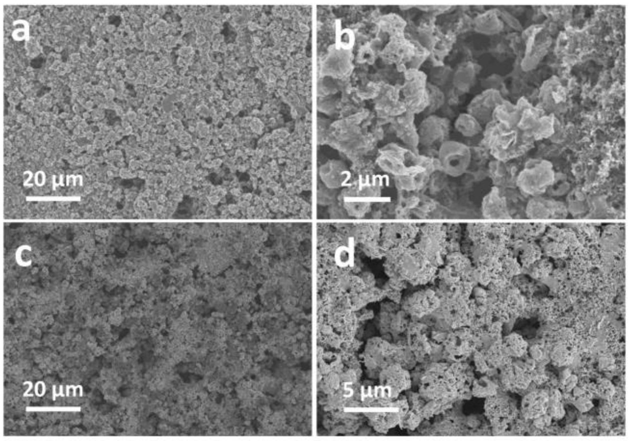

Fig. S21 SEM images of N-Ti_3C_2@CNT microspheres/S cathode (**a, b**) before and (**c, d**) after 100 cycles at 0.2 C

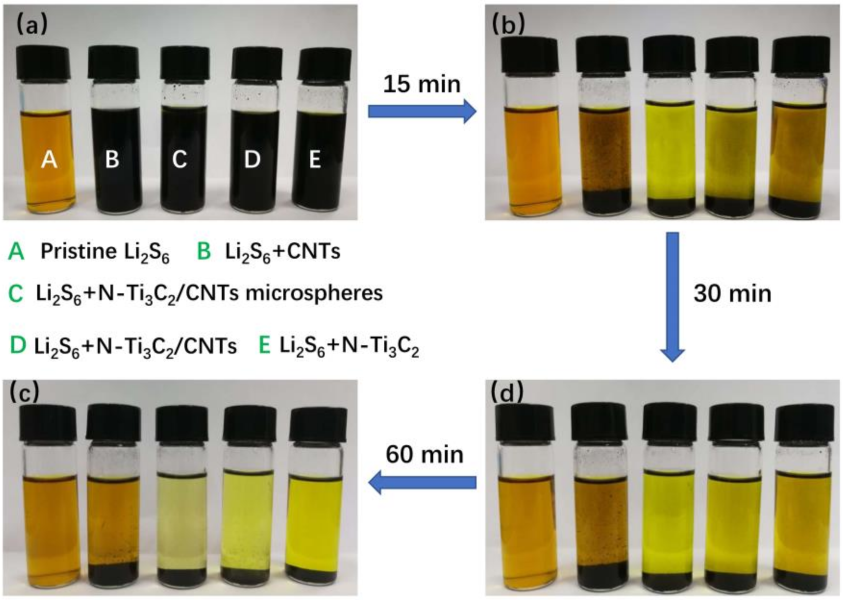

Fig. S22 The adsorption measurements of lithium polysulfides (LiPSs) of commercial CNTs, N-Ti_3C_2@CNT microspheres, N-Ti_3C_2@CNTs and N-Ti_3C_2

Table S1 Specific surface area and pore volume of N-Ti_3C_2, N-Ti_3C_2@CNTs and N-Ti_3C_2@CNT microspheres

Materials	N-Ti_3C_2	N-Ti_3C_2@CNTs	N-Ti_3C_2@CNTs microspheres
Specific surface area (m^2 g^{-1})	263.3	358.4	388.6
Pore volume (cm^3 g^{-1})	0.43	0.66	0.72

Table S2 Elements content analysis of N-Ti_3C_2, N-Ti_3C_2@CNTs and N-Ti_3C_2@CNT microspheres

Materials	C (at%)	Ti (at%)	N (at%)	O (at%)	Ni (at%)
N-Ti_3C_2	48.99	15.44	16.48	19.09	—
N-Ti_3C_2@CNTs	68.11	8.55	10.98	11.56	0.80
N-Ti_3C_2@CNTs microspheres	67.40	8.69	11.86	11.22	0.83

Table S3 Comparison of the cathode performances in this work with other MXene-contained materials reported recently

Sulfur host materials	Sulfur loading (mg cm^{-2})	C-rate (C)	Cycle number	Capacity retention (mA h g^{-1})	Fading rate per cycle (%)	Reference
Mxene nanosheets	1	0.5	650	723	0.05	**1**
3D metal carbide/ mesoporous carbon	2	0.5	300	704	0.14	**2**
Mxene nanosheets	1.2	0.5	500	550	0.062	**3**
Mxene nanosheet/ CNTs composite	1.5	0.5	1200	450	0.043	**4**
Ti_3C_2 nanoribbon	0.7-1	0.5	300	<600	0.24	**5**
Mxene nanosheets/ TiO_2 quantum dots	1.5	2	500	680	0.04	**6**
Mxene nanosheets/ 1T-2H MoS_2-C	1	0.5	300	799	0.07	**7**
Titanium oxide/ Ti_3C_2 hybrids	1	1	1000	662	0.053	**8**
3D porous Mxene/ rGO hybrid aerogels	1.57	1	500	596	0.07	**9**
3D MnO_2 nanosheets/ delaminated-Ti_3C_2	1.2	1	500	501	0.06	**10**
**N-Ti_3C_2@CNTs microspheres**	1.5	1	1000	775	0.016	**This work**
	1.5	4	650	647	0.027	

Supplementary References

[1] X. Liang, A. Garsuch, L.F. Nazar, Sulfur cathodes based on conductive mxene nanosheets for high-performance lithium-sulfur batteries. Angew. Chem. Int. Ed. **54**, 3907-3911 (2015). https://doi.org/10.1002/anie.201410174

[2] W. Bao, D. Su, W. Zhang, X. Guo, G. Wang, 3D metal carbide@mesoporous carbon hybrid architecture as a new polysulfide reservoir for lithium-sulfur batteries. Adv. Funct. Mater. **26**, 8746-8756 (2016). https://doi.org/10.1002/adfm.201603704

[3] J. Song, D. Su, X. Xie, X. Guo, W. Bao, G. Shao, G. Wang, Immobilizing polysulfides with MXene-functionalized separators for stable lithium-sulfur batteries. ACS Appl. Mater. Interfaces **8**, 29427-29433(2016). https://doi.org/10.1021/acsami.6b09027

[4] X. Liang, Y. Rangom, C.Y. Kwok, Q. Pang, L.F. Nazar, Interwoven MXene nanosheet/carbon-nanotube composites as Li-S cathode hosts. Adv. Mater. **29**, 1603040 (2017). https://doi.org/10.1002/adma.201603040

[5] Y. Dong, S. Zheng, J. Qin, X. Zhao, H. Shi, X. Wang, J. Chen, Z.-S. Wu, All-MXene-based integrated electrode constructed by Ti_3C_2 nanoribbon framework host and nanosheet interlayer for high-energy-density Li-S batteries. ACS Nano **12**, 2381-2388 (2018). https://doi.org/10.1021/acsnano.7b07672

[6] X.-T. Gao, Y. Xie, X.-D. Zhu, K.-N. Sun, X.-M. Xie et al., Ultrathin MXene nanosheets decorated with TiO_2 quantum dots as an efficient sulfur host toward fast and stable Li-S batteries. Small **14**, 1802443(2018). https://doi.org/10.1002/smll.201802443

[7] Y. Zhang, Z. Mu, C. Yang, Z. Xu, S. Zhang et al., Rational design of MXene/1T-2H MoS_2-C nanohybrids for high-performance lithium-sulfur batteries. Adv. Funct. Mater. **28**, 1707578 (2018). https://doi.org/10.1002/adfm.201707578

[8] H. Pan, X. Huang, R. Zhang, D. Wang, Y. Chen, X. Duan, G. Wen, Titanium oxide-Ti_3C_2 hybrids as sulfur hosts in lithium-sulfur battery: Fast oxidation treatment and enhanced polysulfide adsorption ability. Chem. Eng. J. **358**, 1253-1261 (2019). https://doi.org/10.1016/j.cej.2018.10.026

[9] J. Song, X. Guo, J. Zhang, Y. Chen, C. Zhang, L. Luo, F. Wang, G. Wang, Rational design of free-standing 3D porous MXene/rGO hybrid aerogels as polysulfide reservoirs for high-energy lithium-sulfur batteries. J. Mater. Chem. A **7**, 6507-6513(2019). https://doi.org/10.1039/C9TA00212J

[10] H. Zhang, Q. Qi, P. Zhang, W. Zheng, J. Chen et al., Self-assembled 3D MnO_2 nanosheets@delaminated-Ti_3C_2 aerogel as sulfur host for lithium-sulfur battery cathodes. ACS Appl. Energy Mater. **2**, 705-714(2019). https://doi.org/10.1021/acsaem.8b01765
